# Effects of PM_2.5_ exposure and air temperature on risk of cardiovascular disease: evidence from a prospective cohort study

**DOI:** 10.3389/fpubh.2024.1487034

**Published:** 2025-01-08

**Authors:** Zhihang Zhang, Ran An, Haoyan Guo, Xuanru Yang

**Affiliations:** ^1^Department of Gynecology, Beijing Hepingli Hospital, Beijing, China; ^2^Chinese Center for Disease Control and Prevention, Beijing, China; ^3^National Institute for Nutrition and Health, Chinese Center for Disease Control and Prevention, Beijing, China; ^4^Graduate School of the First Clinical Medical College, Beijing University of Chinese Medicine, Beijing, China

**Keywords:** air pollution, temperature, PM_2.5_, cardiovascular disease, cohort study

## Abstract

**Background and aims:**

Evidence from extensive cohort studies about the individual and combined associations of air pollution and air temperature with cardiovascular disease (CVD) morbidity is limited. This study aimed to examine the long-term effects of PM_2.5_ exposure and air temperature on CVD based on a cohort study of middle-aged and older populations in China.

**Methods:**

A total of 9,316 non-CVD adults (≥40 years old) who joined the China Health and Retirement Longitudinal Study between 2011 and 2018 were included in our analysis. The two-year average PM_2.5_ concentration and air temperature of the city where participants lived were calculated. The Cox proportional hazards model was conducted to analyze the associations of PM_2.5_ exposure and air temperature with CVD morbidity.

**Results:**

In the multivariable-adjusted model, each 10 μg/m^3^ rise in 2-y PM_2.5_ concentration was associated with an increased risk of developing CVD (hazard ratio [HR]: 1.30; 95% confidence interval [CI]: 1.27–1.32). Compared with individuals in the bearable temperature group, those with low temperatures had a higher risk of CVD (HR: 1.77; 95% CI: 1.53–2.04). Stratified analyses found that cardiovascular metabolic risk factors could not change these associations. Compared with individuals in the low-level PM_2.5_ exposure and bearable temperature group, those in the high-level PM_2.5_ exposure and low-temperature group had a 7.08 times higher risk of CVD (95% CI: 5.55–9.03).

**Conclusion:**

Long-term PM_2.5_ exposure and low air temperature are associated with a higher risk of CVD. Consequently, efforts to reduce air pollution and enhance protection against cold temperatures are vital for mitigating CVD risk.

## 1 Introduction

Cardiovascular disease (CVD) has been highlighted as a significant public health challenge due to its high morbidity, disability, and mortality rates (1). In 2019, the global prevalence of CVD increased to 523 million, and the global deaths from CVD increased to 18.6 million (2). The burden of CVD is expected to rise further, driven by the dual pressures of an aging population and the growing prevalence of metabolic risk factors. Identifying modifiable risk factors is therefore essential to mitigate the adverse effects of CVD.

The detrimental effects of air pollution on CVD have been confirmed by epidemiologic and clinical studies (3–5). Rapid global economic development has resulted in nearly 90% of the world’s population residing in areas with air quality below World Health Organization (WHO) standards (6). As an essential component of air pollution, fine particulate matter mass ≤ 2.5 μm (PM_2.5_) is considered to play a vital role in the risk of developing CVD (7). A meta-analysis showed that every 10 μg/m^3^ increment of PM_2.5_ exposure had a 12% (95% CI: 1.08–1.16) increased risk of CVD mortality (8). In addition, air temperature is also one of the significant risk factors for CVD. At low ambient temperatures, particularly in winter, the risk of CVD rises considerably (9). Hospitalization rates for CVD can also be influenced by air temperature (10, 11). Interestingly, some studies have suggested that temperature might modify the mortality effects of PM_2.5_ (12, 13)_._ A study concerning the effects of daily temperature and daily PM_2.5_ on mortality found that low air temperature significantly enhances the impact of PM_2.5_ on CVD mortality (14).

Existing studies on the effects of air temperature and PM_2.5_ on CVD have focused on short-term effects (15, 16) or cross-sectional studies (17, 18) on CVD mortality (8, 19). There are still significant gaps in our understanding. One of the major gaps is the lack of comprehensive data on how different levels of PM_2.5_ exposure, in combination with various temperature ranges, interact to influence the onset and progression of CVD in different population subgroups. For example, individuals with cardiometabolic risk factors (age, gender, obesity, hypertension, diabetes) may be more vulnerable. Another gap lies in the long—term cumulative effects. While some short—term associations have been explored, the long—term impact of continuous exposure to PM_2.5_ and temperature fluctuations on CVD development is not well-defined.

In this study, we investigated the longitudinal relationship between ambient PM_2.5_ exposure, temperature, and CVD morbidity among Chinese adults using detailed data from the China Health and Retirement Longitudinal Study (CHARLS), considering long-term exposure and analyzing different population subgroups.

## 2 Materials and methods

### 2.1 Study population

The data of our study was extracted from an ongoing cohort study—CHARLS. It is a comprehensive survey conducted in China. The details of the survey have been described elsewhere (20). At baseline, this cohort gathered 17,708 individuals from 28 provinces between 2011 and 2012. Subsequent surveys were conducted in 2013–2014, 2015–2016, and 2017–2018, respectively. Written informed consent was obtained from each patient included in the study, the study protocol conforms to the ethical guidelines of the 1975 Declaration of Helsinki, and the Peking University ethical committees have priorly approved the study protocol. The present study analyzed survey data from 2011 to 2018. Participants under 40 years and those lacking comprehensive general information, BMI, or disease information were disqualified. Ultimately, 15,010 participants were included in our cross-sectional study analyses. We excluded people with CVD at baseline, lost to follow-up, or missing data on CVD information during the 7-year follow-up. Finally, 9,316 subjects were analyzed in follow-up analyses (Supplementary Figure S1).

### 2.2 Exposure measures: PM_2.5_ exposure and air temperature

The annual high-spatial-resolution ground-level PM_2.5_ data was collected from the Atmospheric Composition Analysis Group at Dalhousie University^1^ (21), which relying on NASA’s Sea-viewing Wide Field-of-view Sensor (SeaWIFS), Multiangle Imaging SpectroRadiometer (MISR), and Twin MODerate Resolution Imaging Spectroradiometer (MODIS) to derive aerosol optical depth (AOD) data. These were combined with the GEOS-Chem chemical transport model and ground monitoring data to produce annual PM_2.5_ concentrations at an exceptionally fine spatial resolution of 0.01°*0.01° (960 m*1100 m). The annual PM_2.5_ concentrations in China were estimated at a 1 km × 1 km spatial resolution using a geographically weighted regression model. This model, which achieved an R^2^ of 0.81 for out-of-sample cross-validation, incorporated satellite data, simulated aerosol composition, and land use information. Additionally, the GWR model demonstrated superior performance compared to the global regression model by effectively accounting for spatial heterogeneity. Based on these data, we obtained annual average PM_2.5_ concentrations for the cities where the participants lived from 2010 to 2018. Daily air temperature information during the study period was gathered from China Meteorological Data Sharing Service System.^2^ Participants in the same city were matched to the same yearly mean PM_2.5_ concentration and temperature since we can only obtain the subjects’ city level from CHARLS. We estimated the mean average PM_2.5_ concentrations and temperature during the survey year and the year before. Referring to the previous study (22), the 2-year average PM_2.5_ concentration was calculated as a sign of long-term exposure to PM_2.5_. Besides, winter temperature was defined as the average temperature in January, February, and December. Summer temperature was defined as the average temperature in June, July, and August.

### 2.3 Outcome measurements

Self-reporting was used to gather data on CVD at baseline and in follow-up questionnaires. The participants were classified as having CVD if they responded “yes” to either of the following questions: “Have you been diagnosed with stroke or cardiac events by a doctor?”

### 2.4 Definition of covariates

The covariates in our study include age (continuous), gender (female/male), north or south, rural or urban, marital status (married, unmarried), education (elementary and below, middle, high, and above), drinking status (never, <1 time/month, ≥1 time/month), smoking status (non-smoker, ex-smoker, current smoker), and body mass index (BMI) (continuous). BMI was calculated as weight in kilograms divided by height in meters squared (kg/m^2^). Information about hypertension, diabetes, and dyslipidemia was obtained by asking, “Have you been diagnosed with hypertension, diabetes or high blood sugar, and dyslipidemia by a doctor?” Besides, indoor temperature (hot, bearable, cold) and cooking fuel (clean fuel, solid fuel) were also considered. The types of indoor temperatures were judged by the interviewers. The information on cooking fuel was collected by asking the subjects about the primary source of cooking fuel. If participants answered natural gas, electric, liquefied petroleum gas, or marsh gas, the cooking fuel was categorized as clean fuels. The cooking fuel was classified as solid if participants answered coal, wood, crop residue, solid charcoal, or others.

### 2.5 Statistical analyses

Baseline characteristics were summarized according to CVD status, PM_2.5_, and temperature groups. Continuous variables were presented as means (standard deviations) or medians (interquartile ranges), and categorical variables were presented as numbers (percentages). Differences among groups were compared using the Chi-square test, the variance analysis, or the Kruskal-Wallis test, as appropriate.

In the cross-sectional analyses, binary logistic regression models were used to evaluate the relationship between long-term PM_2.5_ exposure and air temperature with the risk of CVD. The odds ratios (ORs) with 95% confidence intervals (CIs) were calculated by adjusting for age, gender, education, marital status, rural, north, drinking, smoking, BMI, hypertension, diabetes, dyslipidemia, indoor temperature, cooking energy type and air temperature (PM_2.5_). In the longitudinal analyses, the data meet the proportional hazards (PH) assumption (*p* > 0.05), Cox proportional hazards models were used to estimate the individual and combined associations of air pollution and air temperature with CVD morbidity. The hazard ratios (HRs) with 95% CIs were calculated using three models: model 1, unadjusted; model 2, age, gender, education, marital status, rural, north, drinking, smoking, BMI, hypertension, diabetes, and dyslipidemia were adjusted; model 3, indoor temperature, cooking energy type, and air temperature (PM_2.5_) were further adjusted based on model 2. Next, restricted cubic spline with 3 knots for PM_2.5_ and air temperature were further modeled to assess the shape of their associations with incident CVD adjusting covariates. Furthermore, we performed subgroup analyses stratified by cardiometabolic risk factors: age (≤65 and > 65 years), gender, BMI group (<18.5, underweight; 18.5–24, normal; 24–28, overweight and ≥ 28, obesity), hypertension, diabetes, and dyslipidemia. The additive interaction between the effects of air temperature and PM_2.5_ on CVD was also evaluated.

In the cross-sectional and longitudinal analyses, PM_2.5_ exposure and air temperature were used as continuous indicators and categorical variables. We calculated the tertiles of 2-y PM_2.5_ concentration and air temperature. The participants were divided into the lower, middle, and upper groups of PM_2.5_. The participants were divided into the low and bearable air temperature groups using the lowest quartile as the cutoff.

All statistical analyses were performed using R software version 4.1.2. All *p*-values were two-sided, and statistical significance was set at *p* < 0.05.

## 3 Results

### 3.1 Baseline characteristics of study participants

A total of 9,316 participants were included in our longitudinal analysis, among whom the mean age was 58.00 (9.05). In total, 53.54% of participants were female, 84.0% lived in rural, and 44.3% lived in the north. During the 7-year follow-up period, 1,880 participants had developed CVD, resulting in an incidence rate of a 30.89 per 1,000 person-year. Of these cases, 1,419 participants experienced cardiac events, while 643 developed strokes. Table 1 showed that the CVD incidence was higher in females than males. Older people, participants with hypertension, diabetes, and dyslipidemia, and those who used solid fuels for cooking were more likely to develop CVD (*p* < 0.05). Besides, compared to participants without CVD, those who developed CVD were more likely with higher BMI, PM_2.5_ exposure, and lower air temperature (*p* < 0.05). The participant characteristics across the tertiles of 2-y PM_2.5_ concentration were presented in Supplementary Table S1. All variables, except gender, were significantly different among the groups (*p* < 0.05). When participants were grouped by air temperature, most variables showed significant differences between the groups (*p* < 0.05). Compared to the low PM_2.5_ exposure and bearable temperature groups, the high PM_2.5_ exposure and low temperature groups exhibited higher CVD morbidity (Supplementary Tables S1, S2; Supplementary Figure S2).

**Table 1:** The characteristics of the study participants at baseline.

Characteristic	Total (*n* = 9,316)	Non-CVD (*n* = 7,436)	CVD (*n* = 1880)	*p*-value
Age, mean (SD), y	58.00(9.05)	57.44(8.98)	60.19(9.00)	<0.001
Gender (male, n, %)	4,328(46.5)	3,517(47.3)	811 (43.1)	0.001
Rural (n, %)	7,827(84.0)	6,316(84.9)	1,511(80.4)	<0.001
North (n, %)	4,123(44.3)	3,001(40.4)	1,122(59.7)	<0.001
Educational level (n, %)				
Elementary and below	6,388(68.6)	5,086(68.4)	1,302(69.3)	0.183
Middle	2,808(30.1)	2,261(30.4)	547(29.1)	
High and above	120(1.3)	89(1.2)	31(1.6)	
Marital status (married, n, %)	8,368(89.8)	6,722(90.4)	1,646(87.6)	<0.001
Smoking status (n, %)				
Non-smoker	5,785(62.1)	4,590(61.7)	1,195(63.6)	<0.001
Ex-smoker	673(7.2)	507(6.8)	166(8.8)	
Current smoker	2,858(30.7)	2,339(31.5)	519(27.6)	
Drinking status (n, %)				
Never	6,189(66.4)	4,862(65.4)	1,327(70.6)	<0.001
<1 time/month	735(7.9)	609(8.2)	126(6.7)	
≥1 time/month	2,392(25.7)	1965(26.4)	427(22.7)	
BMI, mean (SD), kg/m^2^	23.42(3.54)	23.25(3.46)	24.10(3.76)	<0.001
Hypertension (n, %)	1785(19.2)	1,163(15.6)	622(33.1)	<0.001
Diabetes (n, %)	402(4.3)	261(3.5)	141(7.5)	<0.001
Dyslipidemia (n, %)	645(6.9)	406(5.5)	239(12.7)	<0.001
Cooking fuel (solid fuel, n, %)	5,383(57.8)	4,256(57.2)	1,127(59.9)	0.033
Indoor temperature (n, %)				
Hot	1,140(12.2)	908(12.2)	232(12.3)	0.736
Bearable	7,846(84.2)	6,270(84.3)	1,576(83.8)	
Cold	330(3.5)	258(3.5)	72(3.8)	
2-y PM_2.5_, median (IQR), μg/m^3^	57.63(37.66–74.05)	57.18(37.66–74.05)	61.74(40.87–74.87)	<0.001
2-y air temperature, median (IQR), °C	14.66(12.59–16.76)	15.29(12.79–16.82)	13.47(11.33–15.99)	<0.001

BMI, body mass index; CVD, cardiovascular disease; IQR, interquartile range.

**Figure 1:**
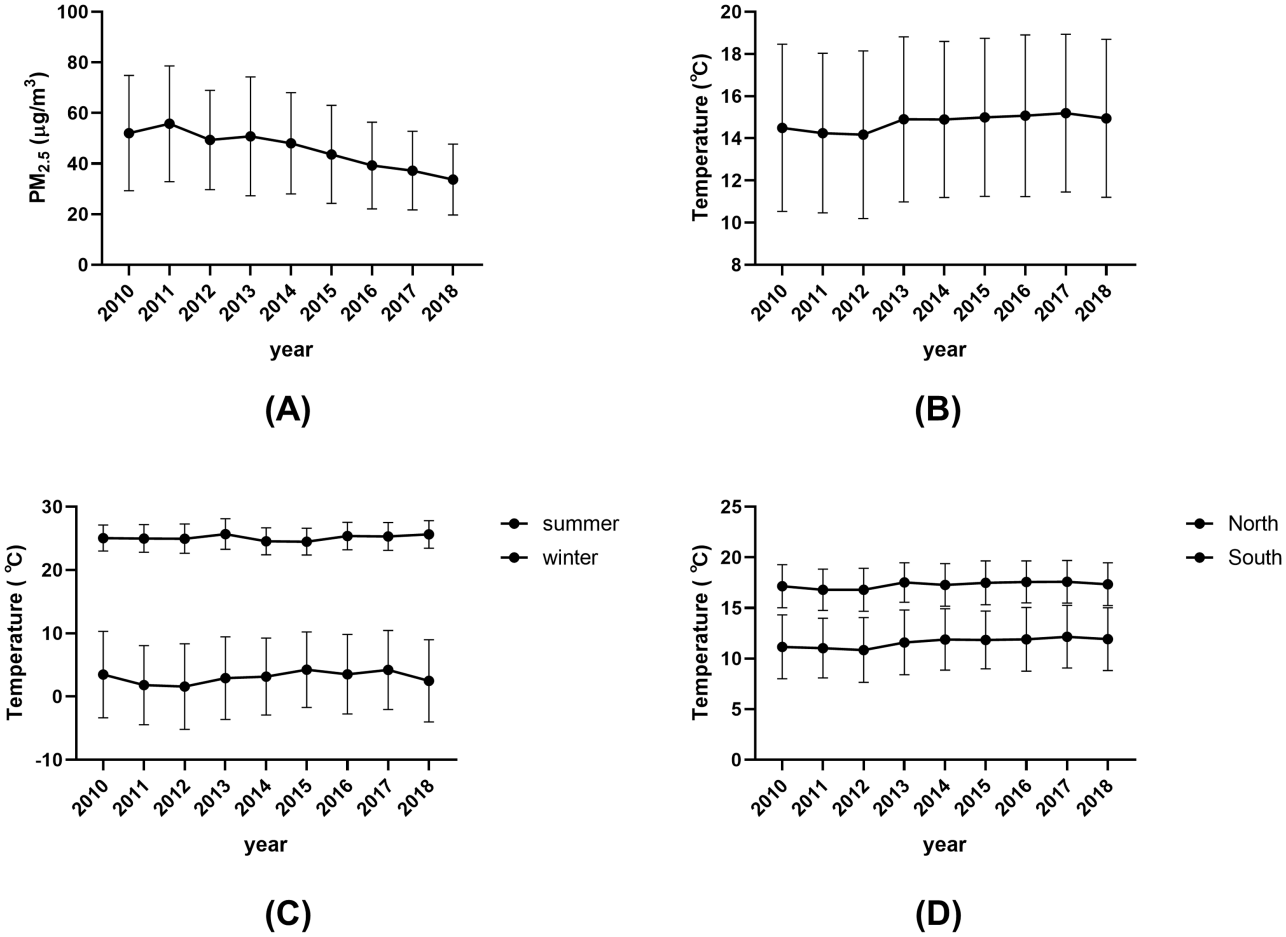
Annual mean levels of PM_2.5_ and air temperature assigned for each subject by calendar year, 2010–2018. **(A)** Annual mean (SD) concentrations of PM_2.5_ from 2010 to 2018. **(B)** Annual mean (SD) temperature from 2010 to 2018. **(C)** Seasonal mean (SD) temperatures for summer and winter from 2010 to 2018. **(D)** Regional mean (SD) temperatures for North and South from 2010 to 2018.

The annual mean (SD) concentrations of PM_2.5_ decreased from 52.06 (22.80) μg/m^3^ in 2010 to 33.71 (14.01) μg/m^3^ in 2018 (Figure 1). The annual mean (SD) temperature ranged from 14.50 (3.97) °C to 14.95 (1.4) °C during the 9 years, showing decreasing trend from 2010 to 2013, and slightly increasing from 2013 to 2018 (Figure 1). Supplementary Figure S3 depicted maps of the 2-year distribution of PM_2.5_ concentration and air temperature for the cities where the participants were lived.

### 3.2 Association between PM_2.5_, temperature, and CVD

**Figure 2:**
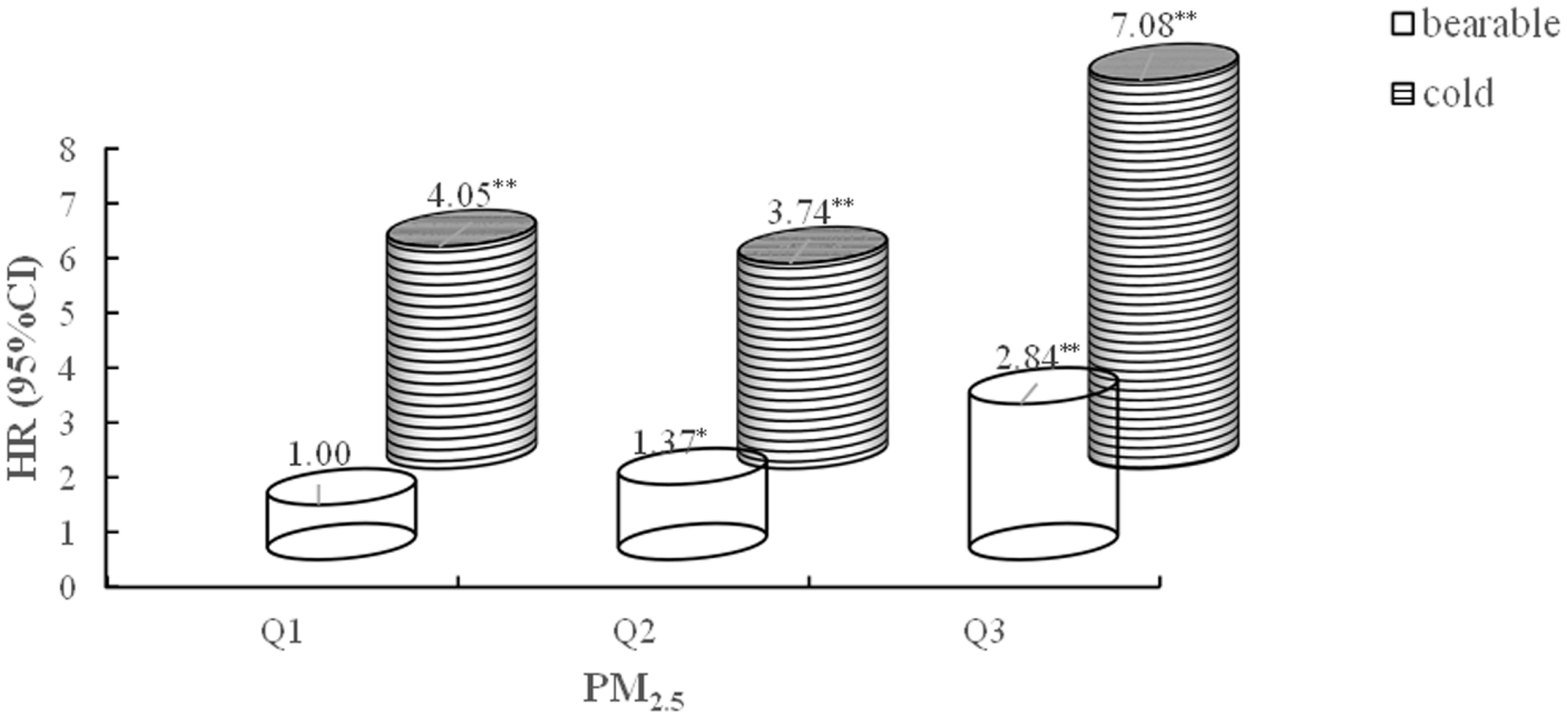
Cox-proportional hazard models for the association between PM_2.5_, air temperature, and CVD, cardiac events, stroke morbidity. Model 1: Unadjusted; Model 2: Adjusted for age, gender, education, marital status, rural, north, drinking, smoking, BMI, hypertension, diabetes, dyslipidemia; Model 3: Model 2 + indoor temperature, cooking energy type, PM_2.5_ **(B)**, and ambient temperature **(A)**. CVD: cardiovascular disease.

In the cross-sectional study analyses, both high-level PM_2.5_ exposure and low air temperature were directly associated with higher CVD prevalence (Supplementary Table S3). In the subsequent longitudinal analyses, these associations were further confirmed. Every 10 μg/m^3^ increase in 2-y PM_2.5_ concentration was associated with a 30% higher risk of developing CVD (HR = 1.30, 95% CI, 1.27–1.32) (Figure 2A). When compared with the Q1 group of 2-y PM_2.5_ concentration, the adjusted HRs for new-onset CVD were 1.13 (1.00–1.28) for Q2 and 1.92 (1.68–2.20) for Q3 (Supplementary Table S4), respectively. The temperature was negatively associated with the risk of CVD morbidity (Figure 2B). Compared with participants in the bearable temperature group, those in the low temperature group had a higher risk of CVD (adjusted HR = 1.77; 95% CI, 1.53–2.04) (Supplementary Table S4). The same tendency was shown in winter and summer temperatures (Supplementary Table S4). The results were consistent in the analyses for cardiac events and stroke incidence (Figure 2; Supplementary Table S4).

We found evidence of nonlinear associations (*P*-nonlinear <0.001) for PM_2.5_ concentration, with strong positive associations with CVD, cardiac events, and stroke incidence at higher concentrations (Supplementary Figures S4A–C). The relationships between air temperature and CVD and stroke were nonlinear (*P*-nonlinear <0.05) while linear for cardiac events (*P*-nonlinear = 0.638) (Supplementary Figures S4E,F). The air temperature had a robust inverse effect on CVD at low to moderate temperatures but weaker at higher temperatures. However, it was shown that a U-shaped association between air temperature and stroke morbidity.

### 3.3 Subgroup analyses

**Figure 3:**
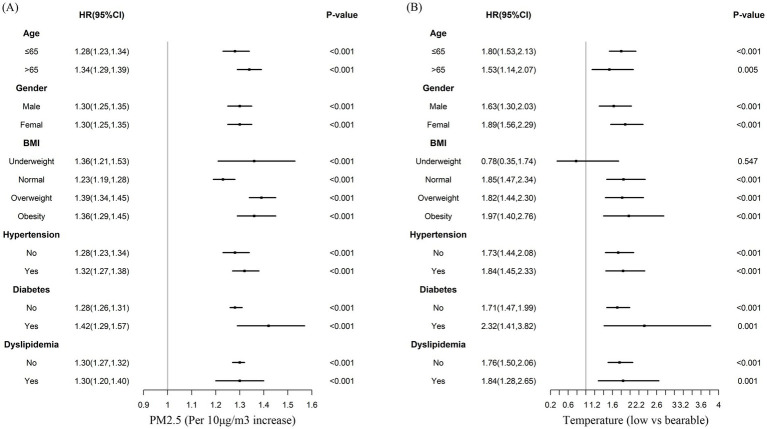
Multivariable-adjusted hazard ratios for the association between PM_2.5_, air temperature, and incident cardiovascular disease by subgroups. Adjusted for age, gender, BMI, hypertension, diabetes, dyslipidemia, education, marital status, rural, north, drinking, smoking, indoor temperature, cooking energy type, and ambient temperature **(A)**, or PM_2.5_ **(B)**.

Subgroups analyses were performed to stratify the association between PM_2.5_, air temperature, and CVD by age, gender, BMI, hypertension status, diabetes status, and dyslipidemia status, as depicted in Figure 3. For PM_2.5_ exposure, the results of the subgroup analyses were consistent with the result of total participants analysis for CVD risk. For air temperature, the results of the subgroup analyses were consistent with the result of total participants analysis for CVD risk, except in the underweight population.

### 3.4 Joint effects of ambient PM_2.5_ and temperature on CVD incidence

**Figure 4:**
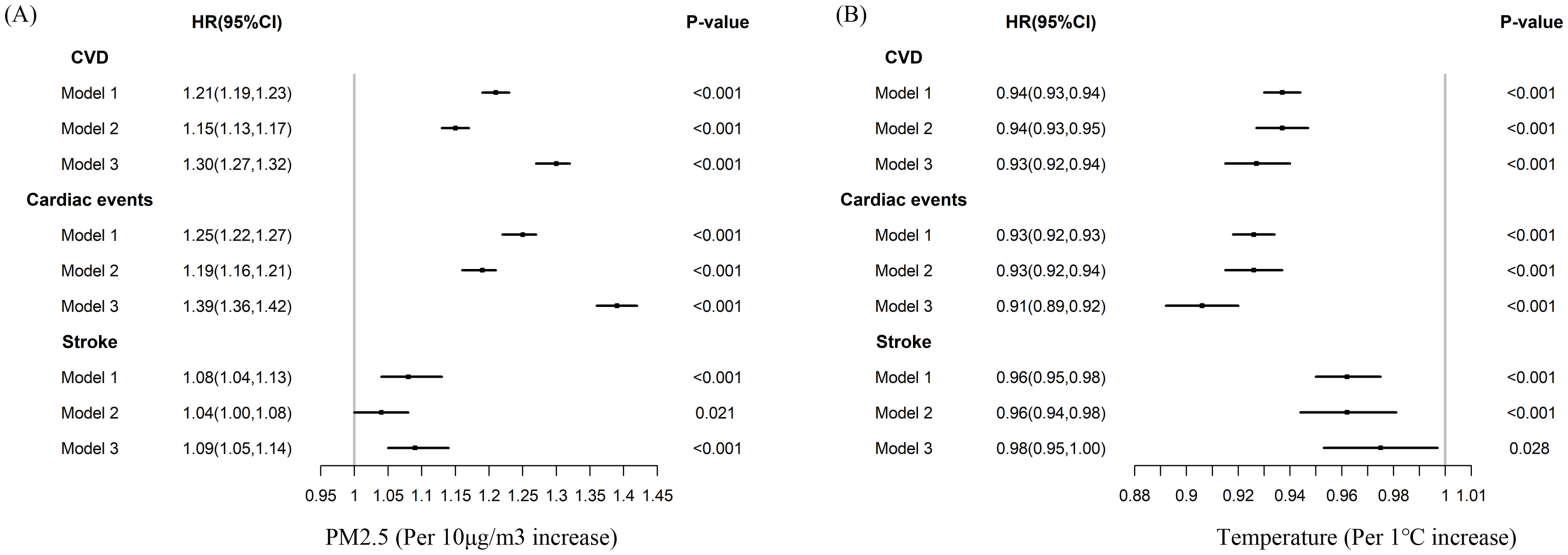
Joint effect of outdoor PM_2.5_ and ambient temperature versus cardiovascular disease incidence. Adjusted for age, gender, education, marital status, rural, north, drinking, smoking, BMI, hypertension, diabetes, dyslipidemia, indoor temperature, and cooking energy type. RERI 2.41 [0.82, 4.00], SI 1.46[1.34, 1.58]. ^*^p < 0.05, ^**^p < 0.0001.

In estimating the joint effects, a significant additive interaction between PM_2.5_ and air temperature on CVD was observed (RERI = 2.41; 95% CI: 0.82, 4.00; SI = 1.46; 95% CI: 1.34, 1.58). Participants in the high PM_2.5_ exposure group (Q3) and low-temperature group faced a 7.08-fold increased risk of developing CVD compared to those in the low PM_2.5_ exposure group (Q1) and bearable temperature group (95% CI: 5.55–9.03) (Figure 4).

## 4 Discussion

Based on the CHARLS survey, this study investigated the individual and combined impacts of ambient PM_2.5_ exposure and air temperature with CVD in the Chinese population for the first time. We presented further proof of the harmful effects of high PM_2.5_ exposure and low temperatures on CVD morbidity. Subgroup analysis showed that age, gender, obesity, hypertension, diabetes and dyslipidemia did not change these association. Furthermore, we discovered that long-term PM_2.5_ exposure and low air temperatures together enhanced the risk of CVD. Therefore, it is necessary for continued air pollution abatement efforts. Better defense against low temperatures may also help reduce the risk of CVD.

The current study found a positive association between long-term PM_2.5_ exposure and the risk of CVD, aligning with existing evidence. Several cross-sectional studies indicate that long-term PM_2.5_ exposure is correlated with an increased likelihood of developing CVD (23, 24). Siregar et al. demonstrated that a 10 μg/m^3^ increase in PM_2.5_ was significantly associated with 29% higher odds of having CVD (OR = 1.29; 95% CI: 1.02, 1.47) among 2,324 participants who were aged ≥40 years old (23). Many studies have reported a positive association between long-term PM_2.5_ exposure and CVD mortality (25–27). Several studies also reported the effects of long-term exposure to PM_2.5_ on CVD events across various populations (28–31). Liang et al. reported that every 10 μg/m^3^ increase in PM_2.5_ exposure increased the risk of CVD incidence (HR = 1.25; 95% CI: 1.22–1.28) and CVD mortality (HR = 1.16; 95% CI: 1.12–1.21) (29). A study using a population-based and nationwide cohort in South Korea also reported that a 10 μg/m^3^ increase in 5-y PM_2.5_ was associated with a 4% higher risk of CVD (95% CI: 0–9%) (32). However, existing studies have not considered the effects of indoor air pollution and temperature on CVD, which may influence the association between PM_2.5_ exposure and CVD risk. In the present study, we supplemented this evidence among the middle-aged and older population in China. Additionally, we identified a nonlinear association between long-term PM_2.5_ exposure and CVD incidence across the entire exposure range, consistent with the findings of Liang et al. (29).

Existing studies have focused on the effects of short-term air temperature on CVD hospitalization rates and CVD mortality (33–35), showing that low or high temperatures were associated with higher rates of CVD hospitalization and mortality. Evidence regarding the long-term effects of air temperature on CVD morbidity is limited. A study conducted on an open cohort of the older adult American population from 2000 to 2016 found that higher average summer temperatures and lower average winter temperatures were associated with an increased risk of CVD hospitalization (36). Our study found that lower annual average air temperatures were associated with a higher risk of CVD. Findings from the China Kadoorie Biobank (CKB) study indicated that lower temperatures increased CVD risk by regulating blood pressure (37). A survey in subtropical southern China showed a U-shaped association between CVD admissions and temperature (34). Our results demonstrated a U-shaped association between air temperatures and stroke morbidity. In contrast, we observed nonlinear associations for CVD morbidity, showing a strong inverse relationship at low to moderate temperatures, but weaker association at higher temperatures. These findings highlight inconsistencies in the effects of higher temperatures on CVD (37). Variations in results may arise from differing exposure estimates or distinct definitions of outcomes.

Previous studies have reported that the association between long-term PM_2.5_ exposure and CVD may be modified by cardiometabolic risk factors (29, 38, 39). In the present study, we explored the modifying effects of age, gender, overweight or obesity, hypertension, type 2 diabetes, and dyslipidemia on the impact of PM_2.5_ exposure and air temperature on CVD incidence. Our findings indicated that the harmful effects of PM_2.5_ exposure and low air temperatures on CVD can be altered by age, gender, and cardiovascular risk factors. Older individuals, those with obesity, hypertension, or diabetes are more susceptible to the cardiovascular risks posed by PM_2.5_ exposure due to various factors. For older adults, a decline in physiological function and the presence of comorbidities make them more vulnerable to inflammation and oxidative stress caused by PM_2.5_. Obesity is linked to a chronic inflammatory state and metabolic disorders, both of which are aggravated by PM_2.5,_ leading to endothelial dysfunction and greater CVD risk. Hypertension causes vascular damage and impaired endothelial function, making blood vessels more susceptible to the harmful effects of PM_2.5_. Diabetes results in vascular and nerve damage as well as abnormal glucose metabolism, which, when combined with PM_2.5_ exposure, increases oxidative stress and inflammation, further heightening the risk of CVD. Several studies hypothesized that cardiometabolic risk factors act as mediators in the relationship between air pollution and CVD which aligns with our results (39, 40). Besides, we detected younger people, females, and those with obesity, hypertension, diabetes, or dyslipidemia were sensitive for the associations between low temperatures and CVD incidence. Studies conducted in two aging populations found that lower air temperatures had detrimental effects on cholesterol levels and blood pressure (41). However, we found that younger individuals, rather than older ones, had a higher risk of developing CVD at low temperatures. One possible explanation is that older individuals are more aware of keeping warm. Additionally, we detected stronger associations between low temperatures and CVD incidence in females. The reasons for the different results by age and sex remain unclear. However, they may be related to lifestyle factors (e.g., the thickness of clothing and outdoor physical activity) and biological differences (e.g., inflammation responses and primary metabolism) (42).

We found a significant additive interaction between high PM_2.5_ exposure and low air temperatures with CVD incidence. Individuals in the highest PM_2.5_ exposure percentile and the lowest air temperature percentile exhibited the highest risk of CVD morbidity. Studies investigating the interaction effects of air pollution and temperature on public health suggest that temperature may modify the association between air pollution and various diseases (14, 43). Although the mechanisms of the influence of PM_2.5_ exposure and temperature on CVD not yet fully understood, there have been several studies addressing this aspect. Several studies hypothesize that the potential mechanisms linking air pollution to CVD include the following: promoting inflammation and oxidative stress (44), mitochondrial dysfunction (45), vascular dysfunction (46), and epigenetic dysregulation of gene expression (47). Current research on possible mechanisms of low temperature-induced CVD involves a variety of physiopathological modulations, including enhanced sympathetic responses to cold (48), activation of the renin-angiotensin system (RAS) (49), and cold-mediated dehydration (50).

Our study further confirmed the harmful effects of high PM_2.5_ exposure and low temperatures on CVD morbidity, which could provide a reference basis for the formulation of public health policies. Firstly, we can provide a scientific basis for setting stricter regulations on PM_2.5_-emitting sources, simultaneously increase keep-warm measures. Secondly, public health campaigns can be designed more effectively. Population who are vulnerable to the effects of high PM_2.5_ exposure and low temperatures on CVD (such as the older adult or those with cardiometabolic risks), targeted educational programs can be developed. These programs can inform these at high-risk groups about the potential risks of high PM_2.5_ exposure and low temperatures and how to reduce their exposure, such as staying indoors during peak PM_2.5_ h and cold weather or using air purifiers.

This study had several advantages. It is the first time that the effects of long-term PM_2.5_ exposure and air temperature on CVD morbidity among Chinese adults were quantified based on a large and representative cohort with 7 years of follow-up. In addition, the modifying effect of cardiometabolic risk factors on these associations was also explored. Furthermore, the association of combined exposures to PM_2.5_ and air temperature with CVD, which has been ignored in other studies, was assessed. Our study had important public health implications, as exposure to PM_2.5_ and low temperature is potentially modifiable, for example, via air conditioning and keeping warm. However, our study is susceptible to some restrictions. First, we examined air temperature and PM_2.5_ exposure at the city level rather than directly measuring pollutant concentrations and temperature at homes, which could cause issues with exposure classification. Second, information on CVD and other disease histories was collected through participants’ self-reports, which might ignore some individuals with CVD. However, we have confirmed whether the answers to the self-reported diseases from the previous survey were correct during each survey, and repeating the confirmation can reduce some bias. In addition, when analyzing the data, we focused on overall trends rather than individual event accuracy. Since our study had a relatively large sample size, the impact of individual inaccuracies in self—reporting on the overall results was likely to be minimized. For example, if a small number of patients misreported CVD events, it would not significantly distort the general patterns of CVD prevalence or associations with other factors in the study population as a whole. Third, prospective cohort studies can be time—consuming and costly. There may be issues with confounding factors not fully accounted for. For example, our study did not control for other air pollution and climate factors confounders, which may affect our results. In addition, the effects of PM_2.5_ exposure and low temperatures on CVD may be influenced by the indoor living environment and time spent outdoors, which were not included in our research. Fourth, the cohort may not be truly representative of the general population, limiting the generalizability of the findings. Last but not the least, the interpretation of results might be over—simplified. Just because an association was found between PM_2.5_ exposure and air temperature on CVD risk, it does not necessarily mean there is a causal relationship. There may be potential mechanisms that have not been fully explored, and further research is needed to confirm them in the future. Finally, during our investigations, we discovered that when we attempted to examine and control for spatially auto—correlation, the performance of our models deteriorated significantly.

## 5 Conclusion

In summary, we discovered that long-term exposure to high-level PM_2.5_ and low air temperatures adversely affected CVD morbidity in Chinese adults, with cardiovascular metabolic risk factors not altering these associations. The combination of high PM_2.5_ exposure and low temperatures significantly enhances the risk of CVD. Our findings are essential for assisting governments in formulating effective public health interventions aimed at addressing environmental pollution and temperature-related health risks.

## Data Availability

The datasets presented in this study can be found in online repositories. The names of the repository/repositories and accession number(s) can be found in the article/Supplementary material.

## Footnotes

^1^ https://sites.wustl.edu/acag/

^2^ http://data.cma.cn/
