# Supplementary material for: Effects of PM2.5 exposure and air temperature on risk of cardiovascular disease: evidence from a prospective cohort study
Source: Front Public Health. 2025 Jan 8;12:1487034. doi: 10.3389/fpubh.2024.1487034 (PMC11750874; doi:10.3389/fpubh.2024.1487034)
Supplement: Supplementary file 1 [file Data_Sheet_1.docx]

Table S1. The characteristics of study participants according to

tertile groups of 2-y PM_2.5_

| Characteristic | Q1  (≤42.56)  (n=3162) | Q2  (42.57-68.99)  (n=3120) | Q3  (≥69.00)  (n=3034) | *P* value |
| --- | --- | --- | --- | --- |
| At baseline | | | | |
| Age, mean (SD), y | 57.81(9.12) | 58.55(9.23) | 57.63(8.76) | <0.001 |
| Gender (male, n, %) | 1455(46.0) | 1473(47.2) | 1400(46.1) | 0.582 |
| Rural (n, %) | 2792(88.3) | 2481(79.5) | 2554(84.2) | <0.001 |
| North (n, %) | 983(31.1) | 836(26.8) | 2804(75.9) | <0.001 |
| *Educational level (n, %)* | | | | |
| Elementary and below  Middle  High and above | 2337(73.9) | 2129(68.2) | 1922(63.3) | <0.001 |
|  | 788(24.9) | 948(30.4) | 1072(35.4) |  |
|  | 37(1.2) | 43(1.4) | 40(1.3) |  |
| Marital status (married, n, %) | 2790(88.2) | 2799(89.7) | 2779(91.6) | <0.001 |
| *Smoking status (n, %)* | | | | |
| Non-smoker  Ex-smoker  Current smoker | 1988(62.9) | 1888(60.5) | 1909(62.9) | <0.001 |
|  | 192(6.1) | 211(6.8) | 270(8.9) |  |
|  | 982(31.1) | 1021(32.7) | 855(28.2) |  |
| *Drinking status (n, %)* | | | | |
| Never  <1 time/month  ≥1 time/month | 2117(67.0) | 2056(65.9) | 2016(66.4) | 0.001 |
|  | 272(8.6) | 204(6.5) | 259(8.6) |  |
|  | 773(24.4) | 860(27.6) | 759(25.0) |  |
| BMI, mean (SD), kg/m^2^ | 22.89(3.47) | 23.18(3.43) | 24.22(3.57) | <0.001 |
| Hypertension (n, %) | 506(16) | 572(18.3) | 7070(23.3) | <0.001 |
| Diabetes (n, %) | 84(2.7) | 120(3.8) | 198(6.5) | <0.001 |
| Dyslipidemia (n, %) | 184(5.8) | 145(4.6) | 316(10.4) | <0.001 |
| Cooking fuel (solid fuel, n, %) | 1885(59.6) | 1780(57.1) | 1718(56.6) | 0.035 |
| *Indoor temperature (n, %)* | | | | |
| Hot  Bearable  Cold | 244(7.7) | 522(16.7) | 374(12.3) | <0.001 |
|  | 2753(87.1) | 2497(80.0) | 2596(85.6) |  |
|  | 165(5.2) | 101(3.2) | 64(2.1) |  |
| At follow-up | | | | |
| New-onset cardiac events (n, %) | 340(10.8) | 387(12.4) | 692(22.8) | <0.001 |
| New-onset stroke (n, %) | 172(5.4) | 193(6.2) | 278(9.2) | <0.001 |
| New-onset CVD (n, %) | 463(14.6) | 539(17.3) | 878(28.9) | <0.001 |

BMI: body mass index; CVD: cardiovascular disease.

Table S2. The characteristics of study participants according to

2-y air temperature groups

| Characteristic | Bearable (>20.27)  (n=6987) | Low ( ≤20.27)  (n=2329) | P |
| --- | --- | --- | --- |
| At baseline | | |  |
| Age, mean (SD), y | 58.38(9.16) | 56.86(8.62) | <0.001 |
| Gender (male, n, %) | 3204(45.9) | 1124(48.3) | 0.044 |
| Rural (n, %) | 5977(85.5) | 1850(79.4) | <0.001 |
| North (n, %) | 1825(26.1) | 2298(98.7) | <0.001 |
| *Educational level (n, %)* | | | |
| Elementary and below  Middle  High and above | 5021(71.9) | 1367(58.7) | <0.001 |
|  | 1898(27.1) | 910(39.1) |  |
|  | 68(1.0) | 52(2.2) |  |
| Marital status (married, n, %) | 6255(89.5) | 2113(90.7) | 0.097 |
| *Smoking status (n, %)* | | | |
| Non-smoker  Ex-smoker  Current smoker | 4405(63.0) | 1380(59.3) | 0.001 |
|  | 508(7.3) | 165(7.0) |  |
|  | 2074(29.7) | 784(33.7) |  |
| *Drinking status (n, %)* | | | |
| Never  <1 time/month  ≥1 time/month | 4600(65.8) | 1589(68.2) | <0.001 |
|  | 515(7.4) | 220(9.5) |  |
|  | 1872(26.8) | 520(22.3) |  |
| BMI, mean (SD), kg/m^2^ | 23.18(3.44) | 24.14(3.71) | <0.001 |
| Hypertension (n, %) | 1236(17.7) | 549(23.6) | <0.001 |
| Diabetes (n, %) | 286(4.1) | 116(5.0) | 0.068 |
| Dyslipidemia (n, %) | 427(6.1) | 218(9.4) | <0.001 |
| Cooking fuel (solid fuel, n, %) | 3911(56.0) | 1472(63.2) | <0.001 |
| *Indoor temperature (n, %)* | | | |
| Hot | 918(13.1) | 222(9.5) | <0.001 |
| Bearable | 5832(83.5) | 2014(86.5) |  |
| Cold | 237(3.4) | 93(4.0) |  |
| At follow-up | | |  |
| New-onset cardiac events (n, %) | 829(11.9) | 590(25.3) | <0.001 |
| New-onset stroke (n, %) | 428(6.1) | 215(9.2) | <0.001 |
| New-onset CVD (n, %) | 1155(16.5) | 725(31.1) | <0.001 |

BMI: body mass index; CVD: cardiovascular disease.

Table S3. Multivariable-adjusted odd ratios for the association between PM2.5, ambient temperature and prevalence of cardiovascular disease

| Exposure | Index | CVD | | Cardiac events | | Stroke | |
| --- | --- | --- | --- | --- | --- | --- | --- |
|  |  | OR (95%CI) | *P*-value | OR (95%CI) | *P*-value | OR (95%CI) | *P*-value |
| PM2.5 | Per 10μg/m3 increase | 1.02(1.00,1.04) | <0.001 | 1.03(1.01,1.05) | <0.001 | 1.02(0.98,1.06) | <0.001 |
|  | Group |  |  |  |  |  |  |
|  | Q1 | Ref. |  | Ref. |  | Ref. |  |
|  | Q2 | 1.04(0.90,1.20) | 0.588 | 0.99(0.85,1.16) | 0.925 | 1.49(1.10,2.02) | 0.011 |
|  | Q3 | 1.35(1.16,1.57) | <0.001 | 1.43(1.21,1.67) | <0.001 | 1.19(0.85,1.66) | 0.316 |
| Temperature | Per 1℃ increased | 0.90(0.89,0.92) | <0.001 | 0.89(0.88,0.91) | <0.001 | 0.97(0.94,1.00) | 0.044 |
|  | Group |  |  |  |  |  |  |
|  | Bearable | Ref. |  | Ref. |  | Ref. |  |
|  | Low | 1.71(1.43,2.05) | <0.001 | 1.63(1.35,1.97) | <0.001 | 1.98(1.30,3.01) | 0.001 |
|  | Winter |  |  |  |  |  |  |
|  | Per 1℃ increased | 0.91(0.90,0.92) | <0.001 | 0.90(0.89,0.91) | <0.001 | 0.97(0.94,1.00) | 0.041 |
|  | Summer |  |  |  |  |  |  |
|  | Per 1℃ increased | 0.93(0.90,0.96) | <0.001 | 0.92(0.89,0.95) | <0.001 | 0.96(0.90,1.03) | 0.305 |

Adjusted for age, gender, education, marital status, rural, north, drinking, smoking, BMI, hypertension, diabetes, dyslipidemia, indoor temperature, cooking energy type and PM_2.5_\ambient temperature; CVD: cardiovascular disease.

Table S4. Multivariable-adjusted hazard ratios for the association between PM2.5, ambient temperature and

incident cardiovascular disease.

| Exposure | Index | CVD | | Cardiac events | | Stroke | |
| --- | --- | --- | --- | --- | --- | --- | --- |
|  |  | HR (95%CI) | *P*-value | HR (95%CI) | *P*-value | HR (95%CI) | *P*-value |
| PM2.5 | Group |  |  |  |  |  |  |
|  | Q1 | Ref. |  | Ref. |  | Ref. |  |
|  | Q2 | 1.13(1.00,1.28) | 0.060 | 1.17(1.01,1.36) | 0.043 | 0.94(0.77,1.16) | 0.587 |
|  | Q3 | 1.92(1.68,2.20) | <0.001 | 2.31(1.98,2.70) | <0.001 | 1.23(0.99,1.53) | 0.061 |
| Temperature | Group |  |  |  |  |  |  |
|  | Bearable | Ref. |  | Ref. |  | Ref. |  |
|  | Low | 1.77(1.53,2.04) | <0.001 | 2.13(1.81,2.52) | <0.001 | 1.11(0.86,1.43) | 0.418 |
|  | Winter |  |  |  |  |  |  |
|  | Per 1℃ increased | 0.95(0.94,0.96) | <0.001 | 0.93(0.92,0.94) | <0.001 | 0.98(0.96,1.00) | 0.075 |
|  | Summer |  |  |  |  |  |  |
|  | Per 1℃ increased | 0.88(0.85,0.90) | <0.001 | 0.85(0.82,0.87) | <0.001 | 0.95(0.91,1.00) | 0.037 |

Adjusted for age, gender, education, marital status, rural, north, drinking, smoking, BMI, hypertension, diabetes, dyslipidemia, indoor temperature, cooking energy type and PM_2.5_\ambient temperature; CVD: cardiovascular disease.

**
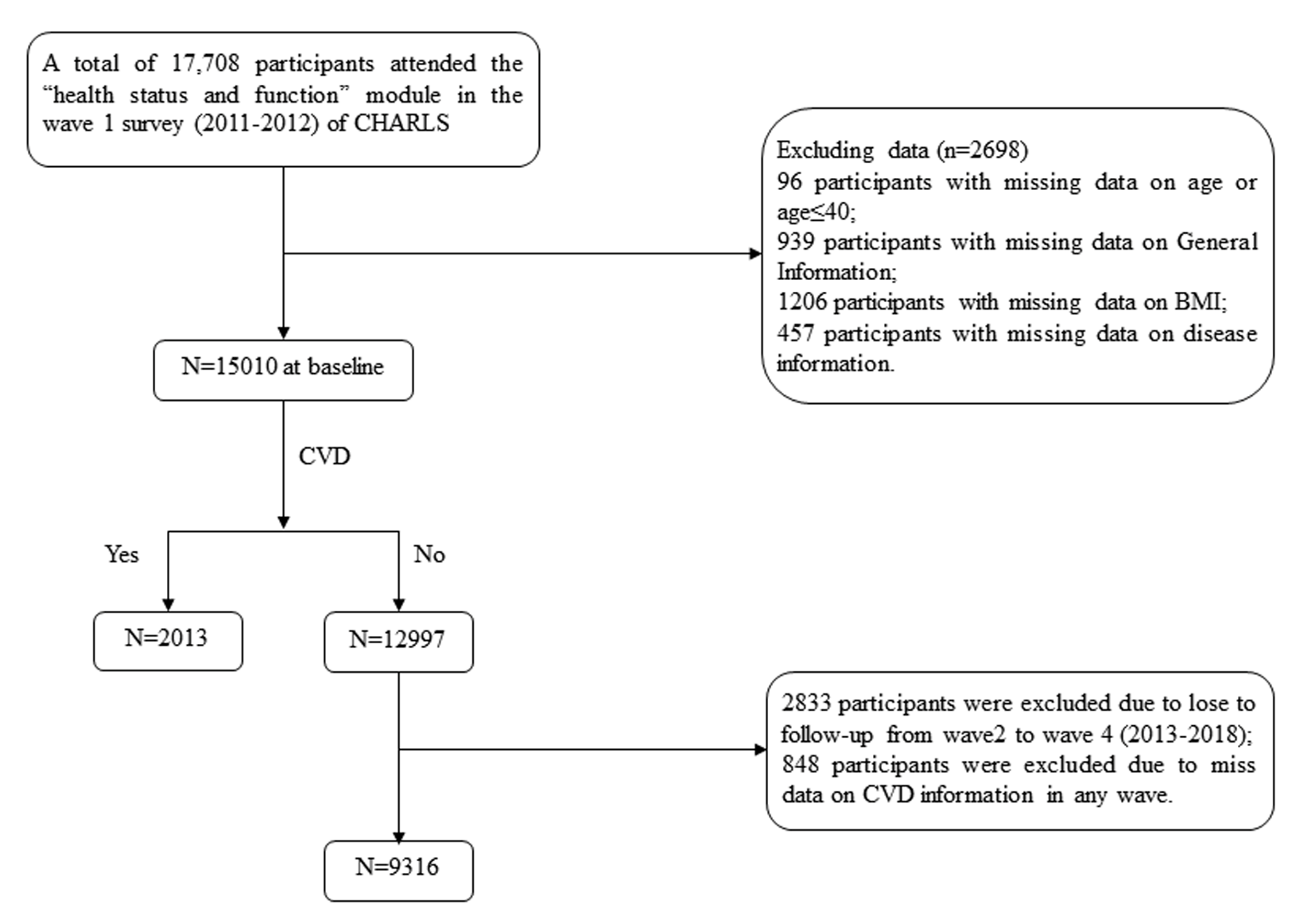
**

Figure S1. Flow chart of our study. CVD: cardiovascular disease; BMI: body mass index.


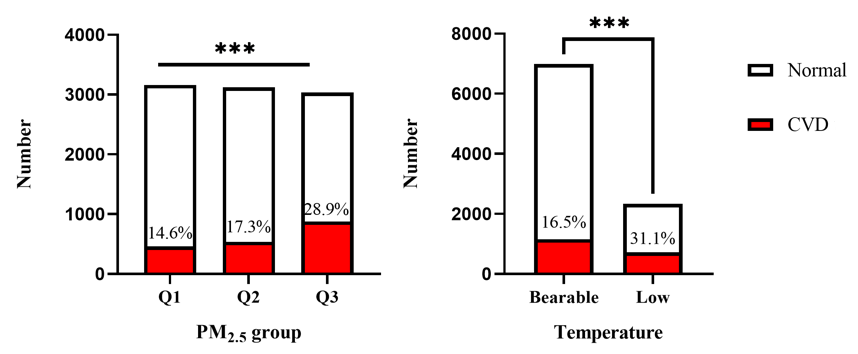


Figure S2. The comparison of the incidence of CVDs according to PM_2.5_ group and temperature group. ****P*＜0.001

CVD: cardiovascular disease.


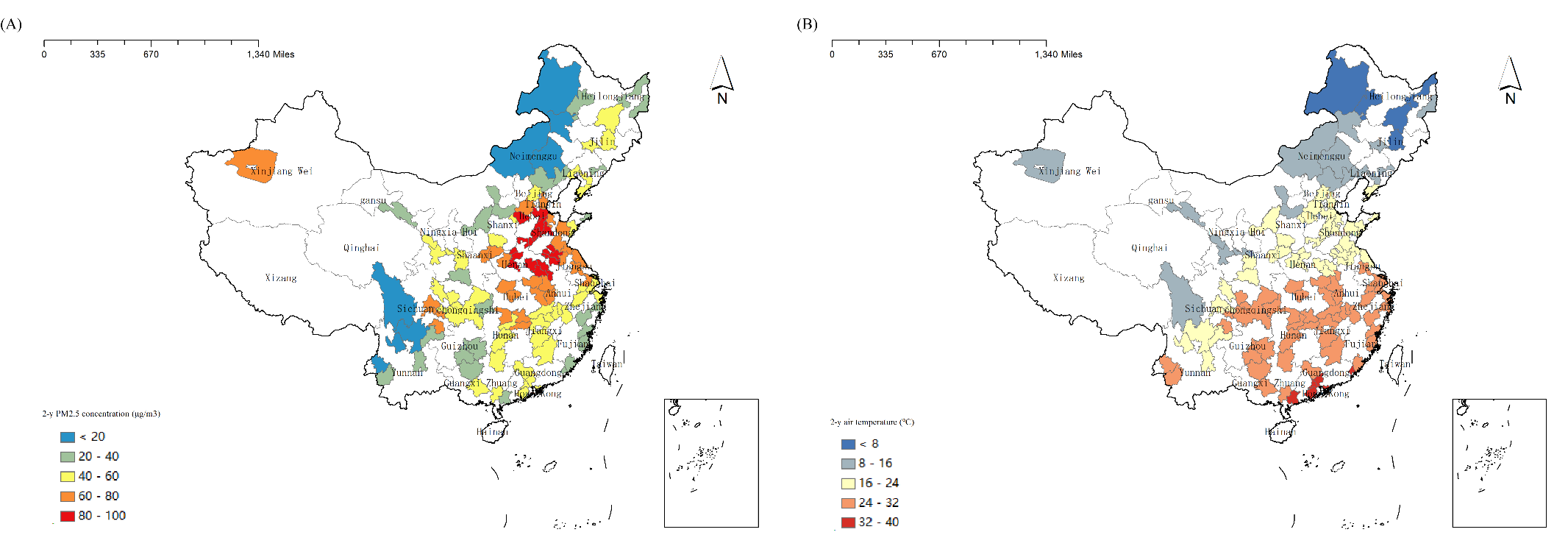


Figure S3. The map of the 2-year distribution of PM_2.5_ concentration and air temperature in the cities in which participants lived.


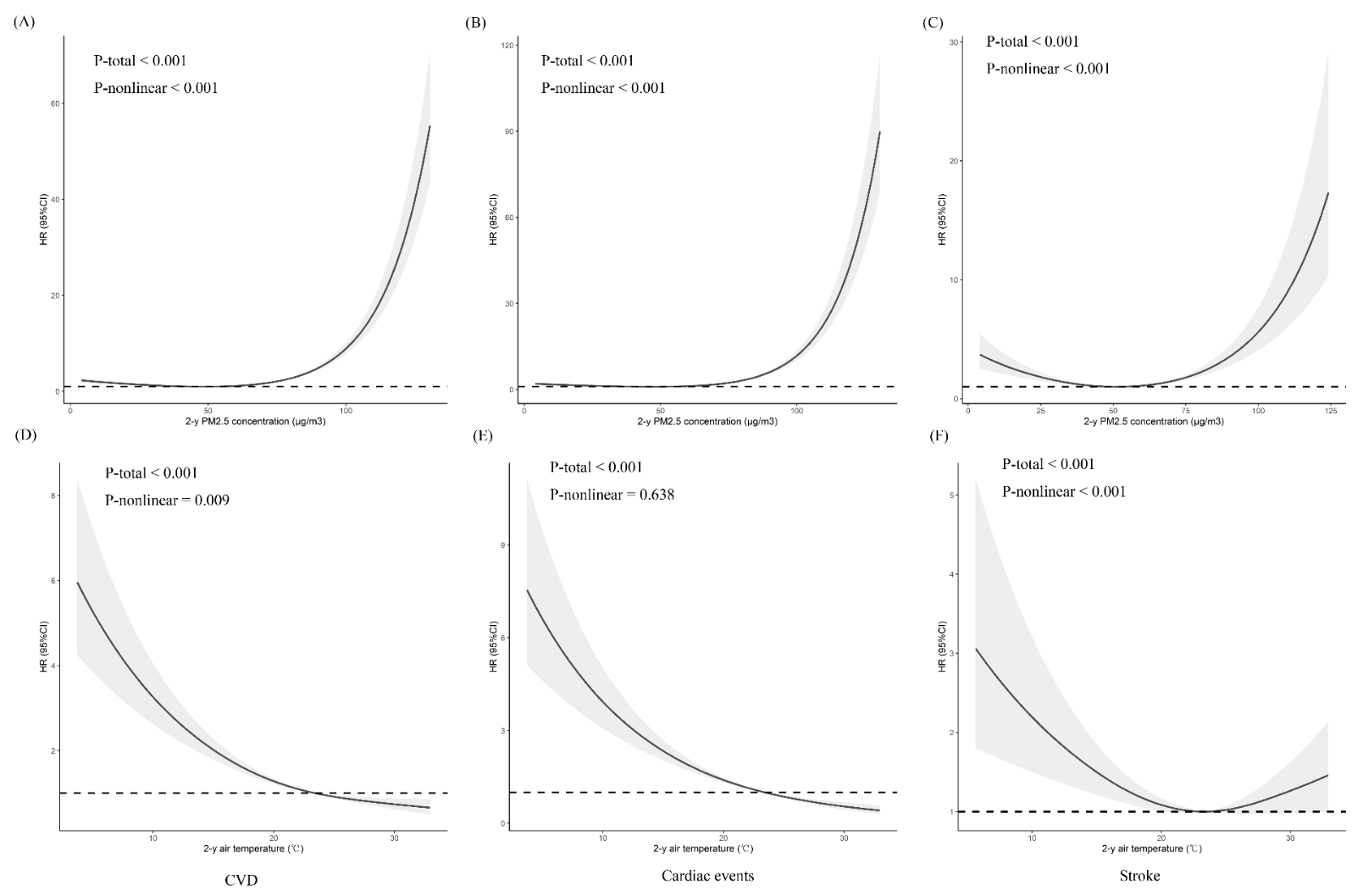


Figure S4. Adjusted cubic spline model of the association between PM_2.5_, air temperature and risk of new-onset CVD, cardiac events and stroke, respectively. Adjusted for age, gender, education, marital status, rural, north, drinking, smoking, BMI, hypertension, diabetes, dyslipidemia, indoor temperature, cooking energy type and PM2.5\ambient temperature; CVD: cardiovascular disease.
